# Loss of p62 Binding Allows TIF‐IA Accumulation in Senescence, Which Promotes Phenotypic Changes to Nucleoli and the Senescence Associated Secretory Phenotype

**DOI:** 10.1111/acel.70334

**Published:** 2025-12-29

**Authors:** Hazel C. Thoms, Tyler S. Brant, Katie Duckett, Yizheng Yang, Jinxi Dong, Hongfei Wang, Freya Derby, Oluwatumilara F. Akeke, Faizah Al‐Alayeen, Amy Newell, Piotr Manasterski, Aishwarya Gopalakrishnan, Derek Mann, Fraser R. Millar, Alex Von Kriegsheim, Juan Carlos Acosta, Fiona Oakley, Lesley A. Stark

**Affiliations:** ^1^ Edinburgh Cancer Research Centre, Institute of Genetics and Cancer (IGC) University of Edinburgh Edinburgh UK; ^2^ Biosciences Institute, Faculty of Medical Sciences Newcastle University Newcastle Upon Tyne UK; ^3^ Instituto de Biomedicina y Biotecnología de Cantabria (IBBTEC), CSIC‐Universidad de Cantabria Santander Spain

**Keywords:** ATM, DNA damage, inflammation, nucleolar, nucleolus, p62, PolI complex, ROS, senescence

## Abstract

A key characteristic of senescent and ageing cells is a reduction in number and increase in size of nucleoli. Although a number of pathways have been suggested, the mechanisms underlying this altered nucleolar phenotype, and the downstream consequences, remain poorly understood. The PolI complex component, TIF‐IA, has previously been implicated in regulating this characteristic nucleolar phenotype in response to stress. Here we explored the role of TIF‐IA in senescence and ageing. We show that TIF‐IA accumulation, particularly in the nucleus and nucleolus, is an early response to oncogene‐ and therapy‐induced senescence (OIS and TIS) in vitro. Using multiple mouse models, we also demonstrate accumulation of TIF‐IA in response to senescence induction and ageing in vivo. We demonstrate that TIF‐IA accumulation is not required for cell cycle arrest but that in OIS and TIS, it is essential for phenotypic changes to nucleoli, the senescence‐associated secretory phenotype (SASP) and establishment of stable senescence. We demonstrate that in proliferating cells, TIF‐IA binds the cargo receptor, p62 (SQSTM1), and that accumulation in senescence occurs as a consequence of ATM activation, which disrupts this interaction. Finally, we show that TIF‐IA accumulation causes an increase in reactive oxygen species (ROS) levels. Together, these results establish TIF‐IA accumulation as a key regulator of the nucleolar phenotype and the SASP in senescence and uncover a novel, p62‐dependent mechanism driving this process. These findings offer significant new insights into nucleolar size regulation in senescence and ageing, and suggest a potential relationship with the inflammatory phenotype.

## Graphical abstract

P62‐TIF‐IA–mediated regulation of senescence and ageing. In healthy tissue, p62 facilitates TIF‐IA turnover. Upon senescence induction, ATM activation increases TIF‐IA phosphorylation at S44, reducing its p62 binding and causing TIF‐IA accumulation. Elevated TIF‐IA drives nucleolar fusion and enhances secretion of senescence‐associated secretory phenotype factors.

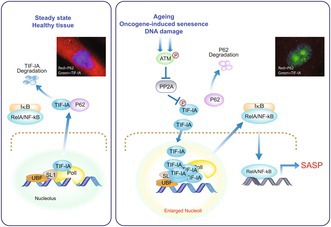

## 1 Introduction

Senescent cells exhibit multiple structural characteristics that contribute to the senescent phenotype. One very striking feature is a reduction in number and an increase in size of the nuclear organelle, the nucleolus (Dillinger et al. 2017; Gutierrez and Tyler 2024; Kasselimi et al. 2022). Nucleoli are membraneless organelles responsible for ribosome biogenesis and the regulation of key cellular processes including cell cycle, metabolism and apoptosis (Lam et al. 2005; Pederson 2018). While proliferating cells generally have multiple small nucleoli, cells triggered to undergo senescence have one prominent, enlarged nucleolus (Dillinger et al. 2017; Gutierrez and Tyler 2024; Kasselimi et al. 2022; Tiku and Antebi 2018). We have named this the senescence‐associated nucleolar phenotype (SANP). Increased nucleolar size is also a hallmark of chronological ageing and is observed in patients with the ageing disorder, Hutchinson‐Gilford progeria syndrome (Buchwalter and Hetzer 2017; Gutierrez and Tyler 2024; Tiku et al. 2016). Nucleoli are increasingly recognised as biomolecular condensates that form as a result of phase separation (Brangwynne et al. 2011) and so it has been proposed that the rapid reduction in number and increase in size of nucleoli in senescence and ageing is a consequence of coalescence of nucleolar droplets (Caragine et al. 2019; Dillinger et al. 2017). However, the mechanisms by which nucleolar phenotype is regulated, and the downstream consequences of these changes in ageing, remain poorly understood.

One protein we and others have shown to play a key role in regulating nucleolar phenotype in response to stress is TIF‐IA (RRN3). The rate limiting step of ribosome biogenesis is transcription of ribosomal DNA (rDNA), which is mediated by the RNA polymerase I (PolI) complex (Drygin et al. 2010). TIF‐IA is an essential component of this complex as it recruits PolI to the rDNA promoter (Jin and Zhou 2016). It is also the component of the complex reported to transduce environmental signals to the PolI transcriptional machinery. Nutrient starvation and specific stresses alter TIF‐IA phosphorylation, reducing nucleolar size (Mayer et al. 2005). In contrast, our research has shown that ceramide and stresses such as UV‐C radiation and aspirin lead to TIF‐IA degradation, which causes a significant increase in nucleolar size and a reduction in nucleolar number (Chen et al. 2018). Given this stress‐mediated nucleolar phenotype mirrors the SANP, here we explored the role of TIF‐IA in regulating nucleolar phenotype in senescence.

We show that TIF‐IA accumulates as an early response to oncogene and therapy‐induced senescence (OIS and TIS) in vitro, and in response to OIS induction and ageing in vivo. We demonstrate that depletion of TIF‐IA inhibits the SANP, the senescence‐associated secretory phenotype (SASP), and established senescence in OIS and TIS models, while overexpression of TIF‐IA mimics the effects of OIS and TIS induction on the SANP and SASP. We show that the p62 cargo receptor facilitates TIF‐IA degradation in proliferating cells and that accumulation in senescence occurs downstream of DNA damage and ATM activation, which blocks the TIF‐IA–p62 interaction. We conclude from these data that TIF‐IA accumulation is a key regulator of the nucleolar phenotype and the SASP in senescence and propose a novel mechanism responsible for this accumulation. These data have significant relevance to ageing and disease.

## 2 Methods

### 2.1 Cell Lines and Treatments

IMR90 human primary fibroblasts stably expressing a 4‐hydroxytamoxifen‐inducible HRas^G12V^‐fusion protein (ER:Ras) or a negative control vector (ER:Stop) were a kind gift from Professor JC Acosta (IBBTEC, University of Cantabria) and have been described elsewhere (Innes and Gil 2019). A549 WT and A549‐ATG5^−/−^ (∆*ATG5*) cells were a kind gift from Professor Simon Wilkinson (Institute of Genetics & Cancer, University of Edinburgh). WT IMR90 fibroblasts and HCT116 colon cancer cells were obtained from the ATCC. All cells were grown in DMEM (Gibco) supplemented with 10% foetal bovine serum and 1% penicillin/streptomycin and were maintained at 37°C in a humidified atmosphere with 5% CO_2_. Treatments were performed under the same conditions. 4‐Hydroxytamoxifen (4‐OHT) and etoposide were obtained from Sigma Aldrich, and BrdU was purchased from Abcam.

### 2.2 Mouse Models of Ageing and Senescence

Dr. Fraser Millar (Institute of Genetics & Cancer, Edinburgh) kindly provided formalin fixed, paraffin embedded (FFPE) liver sections from C57BL/6 mice induced to express either oncogenic Ras (Nras^G12V^) or an inactive mutant (Nras^G12V/D38A^) using transposon‐mediated intrahepatic gene transfer via a tail vein injection of ‘Sleeping Beauty’ plasmids. Tissue was collected and processed 6 days after tail vein injection. Full details can be found in Millar et al. (2022). FFPE sections from the colon of young (3–4 months) and old (16–17 months) C57BL/6 wild type (WT) or *nfkb1* ^−/−^ mice were kindly provided by Professor Fiona Oakley (Liver Fibrosis Group, Newcastle University). *nfkb1* ^−/−^ mice show signs of chronic inflammation and premature ageing from approximately 9 months of age (Jurk et al. 2014).

All mice were housed in pathogen‐free conditions, kept under standard conditions with a 12‐h day/night cycle and free access to food and water. All experiments were approved by the relevant body (Biomedical Research Facility, University of Edinburgh or Newcastle Ethical Review Committee) and performed under a UK Home Office licence in accordance with the ARRIVE guidelines.

### 2.3 Immunohistochemistry

Liver or colon sections (3 μm thickness) were dewaxed, rehydrated and stained using standard DAB immunohistochemistry procedures. Sections were incubated overnight at 4°C with the primary antibodies: rabbit RRN3 (Abcam 251933) and mouse NRas (Santa Cruz Biotechnology sc‐31), diluted 1:500 in antibody diluent (Dako). Stained slides were scanned using the NanoZoomer XR slide scanner (Hamamatsu) with NDP Scan v3.4 software (Hamamatsu) then analysed using QuPath Version 0.4.3 software (Bankhead et al. 2017). The number of TIF‐IA positive cells (liver sections) or cytoplasmic and nuclear TIF‐IA intensity (colonic sections) was quantified for 5 equal, randomly selected regions of interest (ROI) per section. Data per ROI was generated. Pooled data are presented.

### 2.4 Immunocytochemistry BrdU and ROS Assays

Cells were grown on glass coverslips, treated as stated then immunocytochemistry performed using standard procedures (Stark and Dunlop 2005). Fixation was carried out with paraformaldehyde and permeabilisation with Triton X‐100. Primary antibodies used were rabbit TIF‐IA (1:200, Assay BioTech B8433), mouse C23 (1:200, Santa Cruz Biotechnology sc‐8031) and mouse p62 (1:100, BD Transductions Labs, BD610833). Alexa Fluor‐conjugated secondary antibodies (Invitrogen) were utilised. Cells were mounted in Vectashield with DAPI for visualisation of nuclei. Bromodeoxyuridine (BrdU) incorporation was used to monitor cell proliferation. Briefly, cells were treated with 10 μM BrdU for 18 h prior to fixation and immunocytochemical staining with an anti‐BrdU antibody (Invitrogen BU‐1). CellRox Deep Red (Invitrogen) was used to identify and quantify levels of reactive oxygen species. Cells were transfected with pEGFP‐C1 or pEGFP‐C1‐TIF‐IA for 24 h as detailed below. They were then incubated with 5 μM CellRox Deep Red reagent for 30 min prior to fixation. Nuclei were stained with DAPI as above. All images were visualised with a Zeiss Axio Imager M2 microscope equipped with an LED light source and captured using an ORCA‐Flash 4.0 LT camera (Hamamatsu) with MicroManager software to control exposures across the different fluorescent channels. All images per experiment were captured using a constant exposure time for each channel.

### 2.5 Image Quantification

Image analysis was conducted using either FIJI (1.54f) or Cell Profiler (4.2.5) software as specified in figure legends. To quantify nuclear or nucleocytoplasmic ratios of TIF‐IA using FIJI, signal intensity was determined in a defined area of the nucleus (indicated by DAPI) and an equal area at the subnuclear periphery (cytoplasm). To quantify nuclear or nucleocytoplasmic ratios using Cell Profiler, nuclei were identified in the DAPI channel using the identify primary objects module. The primary object was expanded by 50 pixels to generate a secondary object. TIF‐IA intensity was then measured in the nuclei (primary object) and the cytoplasm (primary object subtracted from the secondary object). Nucleolar area was quantified in FIJI using manual segmentation. In fixed cells, nucleoli were defined as regions devoid of DAPI staining, as previously described (Chen et al. 2018). In phase contrast images, nucleoli were defined as nuclear regions with an altered refractive index (dark circles). Cells exhibiting Senescence‐Associated Heterochromatic Foci (SAHF) were counted on fixed images as described elsewhere (Noren Hooten and Evans 2017). For all image analysis, at least 5 random fields of view were quantified per condition for three independent experiments or as specified in the text. The minimum number of cells analysed is given in each figure legend.

### 2.6 Plasmids, siRNA and Transfections

GFP‐tagged WT p62, p62 ∆PB1 and p62 ∆UBA have been described previously (Lobb et al. 2021). pEGFP‐C1‐TIF‐IA WT was kindly gifted by Proffesor Ingrid Grummt (German Cancer Research Centre, Heidelberg). pEGFP‐C1‐TIF‐IA S44A and S44D mutants were generated in house and have been described elsewhere (Chen et al. 2018). SignalSilence SQMTS1/p62 siRNA was obtained from Cell Signalling Technologies. siRNA to TIF‐IA was custom made TIF‐IA#1 CUAUGUAGAUGGUAAGGUU; TIF‐IA#2 CUAGAAUUCCGUUCUUCUA; Control AGGUAGUGUAAUCGCCUUG (Chen et al. 2018). Plasmids and siRNA were transfected into cells using Lipofectamine 2000 (Invitrogen) according to the manufacturer's instructions 24 h prior to any other treatment. For longer term knockdown, transfections were repeated as described in the text.

### 2.7 Quantitative RT‐PCR (qRT‐PCR)

RNA was extracted from cells using the RNeasy mini kit (Qiagen) following the manufacturer's instructions. Extracted RNA was purified using RQ1 RNase‐free DNase (Promega) then cDNA generated using M‐MLV reverse transcriptase and random primers (Promega). Taqman (Thermo Fisher Scientific) or SYBR Green (Roche) assays and a LightCycler 480 system were used to quantify transcript levels. For studies using qRT‐PCR to explore the causal role of ROS in the SASP, cells were pretreated with the ROS scavenger, NAC (*N*‐acetyl‐l‐cysteine) (5 mM) for 24 h prior to 24 h transfection with EGFP‐C1 or EGFP‐C1‐TIF‐IA. Primers were supplied by Integrated DNA Technologies for the following sequences: *IL‐1A For* AGTGCTGCTGAAGGAGATGCCTGA; *IL‐1A Rev* CCCCTGCCAAGCACACCCAGTA; *IL‐6 For* CCAGGAGCCCAGCTATGAAC; *IL‐6 Rev* CCCAGGGAGAAGGCAACTG; *IL‐8 For* GAGTGGACCACACTGCGCCA; *IL‐8 Rev* TCCACAACCCTCTGCACCCAGT; *MMP1 For* AAAGGGAATAAGTACTGGGC; *MMP1 Rev* CAGTGTTTTCCTCAGAAAGAG; *MMP3 For* TAAAGACAGGCACTTTTGG; *MMP3 Rev* GAGATGGCCAAAATGAAGAG; *NFKB1A For* TCCACTCCATCCTGAAGGCTAC; *NFKB1A Rev* CAAGGACACCAAAAGCTCCACG *TIF‐IA For* GTTCGGTTTGGTGGAACTG; *TIF‐IA Rev* CGGAATTCTAGCAGCCAGT. The Comparative *C*_T_ method (or ∆∆*C*_T_ method) was used for calculation of relative gene expression.

### 2.8 Senescence‐Associated β‐Galactosidase Staining

Levels of senescence were determined by examining the extent of β‐galactosidase activity. Briefly, the cells were fixed with 4% glutaraldehyde in PBS (Sigma Aldrich) for 10 min at room temperature. They were then washed with PBS, pH 6.0, and incubated with staining solution (1 mg/mL X‐Gal [Melford] with 5 mM potassium ferrocyanide [Sigma Aldrich] and 5 mM potassium ferricyanide [Sigma Aldrich] in PBS) for 16 h at 37°C in the dark. Following incubation, the cells were washed with PBS and the percentage of cells staining positive for β‐galactosidase was determined manually by light microscopy. At least 50 cells were counted from 10 randomly selected fields of view per group per replicate. To monitor β‐galactosidase activity in naïve HCT116 cells following transfer of conditioned medium, HCT116 cells were transfected with pEGFP control or pEGFP‐C1‐TIF‐IA as above. Forty‐eight hours later, cells were imaged to confirm efficient transfection then conditioned medium was removed and applied to naïve HCT116 cells for 8 days. β‐galactosidase activity was then determined in the naïve cells as above.

### 2.9 Cell Lysis and Western Blot Analysis

Whole cell extracts were prepared and protein quantified using Bradford Assays (BioRad), as previously described (Stark and Dunlop 2005). Western blots were carried out using standard protocols as previously described (Stark and Dunlop 2005). Primary antibodies used were: TIF‐IA (Rabbit, 1:1000, BioAssayTech B8433); Rrn3 (Mouse, 1:500, Santa Cruz sc‐390,464); p62 (Mouse, 1:1000 BD Transduction Labs BD610833); GFP (Rabbit, 1:1000 Santa Cruz, sc‐8433); pATM (Rabbit, 1:500 phospho‐ATM (Ser1981) (D6H9) Cell Signalling Technology 5883); Actin‐HRP (Mouse 1:2000 sc‐47778 Santa Cruz). Bands were quantified using FIJI as per software instructions.

### 2.10 Immunoprecipitation Assays

Endogenous TIF‐IA and p62 were immunoprecipitated from 1 mg whole cell lysate using mouse TIF‐IA antibody (Santa Cruz Biotechnology) and protein G Dynabeads (Invitrogen), as previously described (Chen et al. 2018). IgG acted as a control. To immunoprecipitate GFP‐tagged proteins, cells were transfected with the relevant plasmids, treated as described then GFP‐Trap beads (Chromotek) were used to isolate GFP‐tagged proteins from whole cell lysates. Bound proteins were resolved by SDS polyacrylamide gel electrophoresis then analysed by western blot analysis.

### 2.11 TIF‐IA Interactome

Etoposide effects on the TIF‐IA interactome were analysed by quantitative mass spectrometry as previously described (Turriziani et al. 2014). Briefly, HCT116 cells were treated with DMSO or etoposide (100 μM) for 8 h. Cells were then pelleted, disrupted in NP40 buffer and endogenous TIF‐IA immunoprecipitated as above. Tryptic peptides were generated by on bead digestion and analysed on a Q‐Exactive mass spectrometer connected to an Ultimate Ultra3000 chromatography system (both Thermo Fisher Scientific, Germany). Mass spectra were analysed using the MaxQuant Software package in biological triplicate and technical replicate. Fold change for each condition was determined by comparing peptide abundance in IgG versus mouse TIF‐IA antibody. Fold change and moderated *p* values were generated using the data from three replicates.

### 2.12 Statistical Analyses

GraphPad Prism Software (version 9) was used for statistical analysis. Normality tests were performed on all datasets to determine data distribution, then the relevant parametric or nonparametric test used (as outlined in figure legends) to determine significance. For cell imaging experiments, intensity/size values for all cells analysed in all repeats were pooled, outliers identified and removed with Prism Software using ROUT with an aggressive parameter (*q*) of 1% (middle of the range). Statistics were then performed. For analysis of mouse sections, Tukey's multiple comparison test was performed on QuPath data from five ROI per mouse per condition (see above). For quantitative PCR, *p* values were generated by comparing three technical repeats, for three individual experiments, or as specified in the figure legend. The number of repeats for all experiments is given in the figure legends and the exact *p* values specified. Data were deemed to be significant if *p* < 0.05.

## 3 Results

### 3.1 TIF‐IA Accumulates as an Early Response to Oncogene and Therapy‐Induced Senescence

**FIGURE 1:**
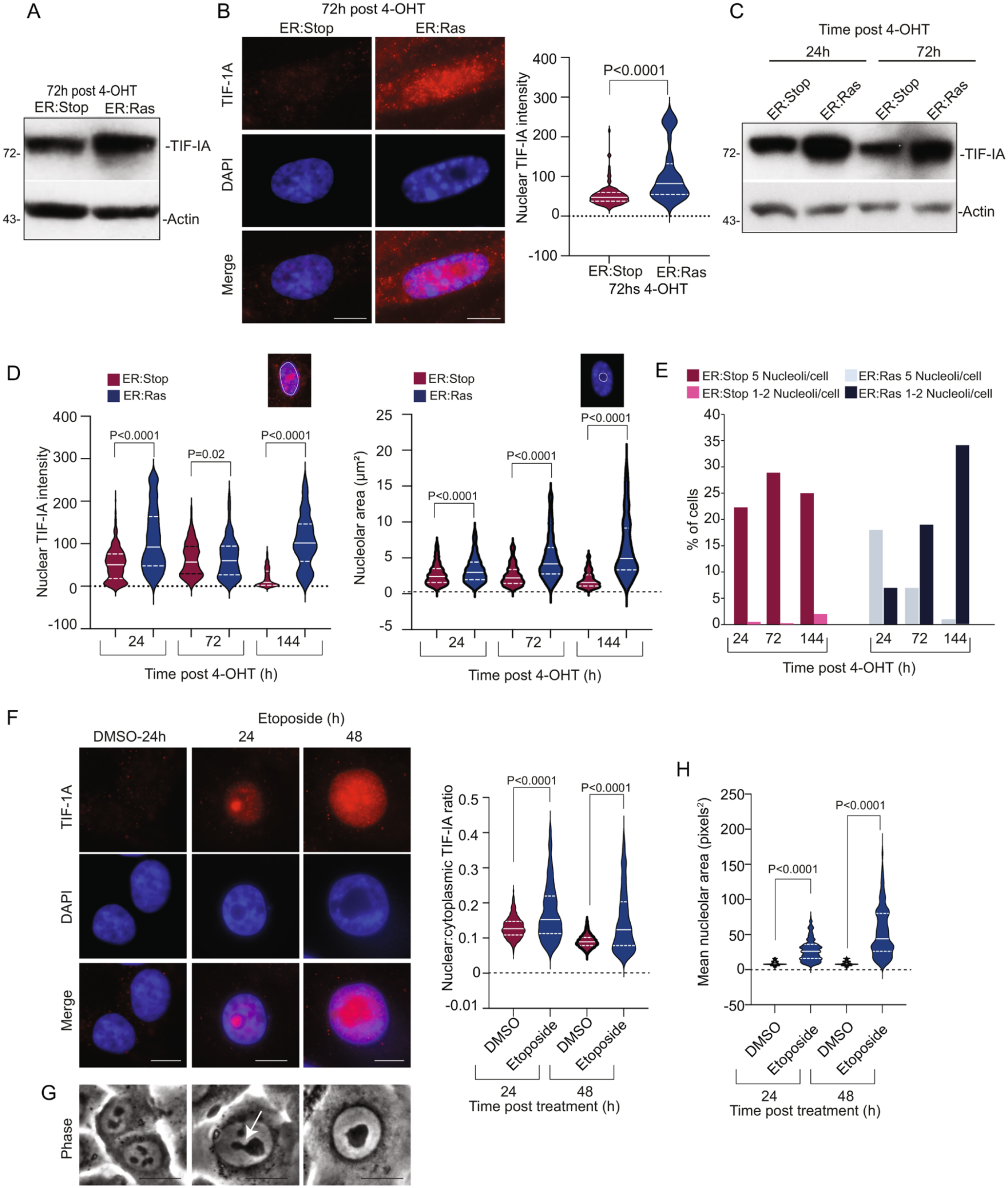
TIF‐IA protein accumulation is an early event in oncogene and therapy‐induced senescence that parallels nucleolar fusion. (A–D) TIF‐IA accumulation is an early response to oncogene‐induced senescence (OIS). IMR90 ER:Ras cells were treated with 4‐hydroxytamoxifen (4‐OHT) for the times specified to induce oncogenic Ras expression. IMR90 ER:Stop cells serve as a control and retain proliferative capacity with 4‐OHT. (A) Anti‐TIF‐IA immunoblot (sc‐390464) performed on whole cell lysates (B) Immunocytochemistry was performed on ER:Stop and ER:Ras cells 72h post 4‐OHT exposure (*n* = 3). Left: Representative immunomicrographs. Right: Cell Profiler quantification of nuclear TIF‐IA. Violin plots show pooled data for three experiments (minimum 100 cells per group). (C–E) Levels and localisation of TIF‐IA were monitored in ER:Stop and ER:Ras cells exposed to 4‐OHT in time course studies. (C) Immunoblot performed on whole cell lysates as above shows TIF‐IA increases at 24 h. See Figure S1C for quantification (*n* = 3) (D, E) Anti‐TIF‐IA immunocytochemistry was performed (*n* = 3). See Figure S1D for representative images. (D) FIJI software was used to quantify nuclear TIF‐IA intensity and nucleolar area (manual segmentation of areas devoid of DAPI). Violin plots show pooled data for three experiments (minimum 275 cells per group). (E) The number of nucleoli per cell was determined using area devoid of DAPI. Graph shows the percentage of cells with 5 or 1–2 nucleoli per time point. (F–H) TIF‐IA accumulation is an early response to therapy‐induced senescence. HCT116 cells were treated with DMSO (carrier control) or etoposide (100 μM) for the times specified in hours (h) (*n* = 3) (F) Left: Representative immunomicrographs showing nuclear accumulation of TIF‐IA in response to etoposide. Right: Cell Profiler was used to quantify nucleocytoplasmic ratios of TIF‐IA. Violin plots show pooled data for three experiments (minimum 280 cells per group). (G) Representative phase contrast images (×40 magnification) of live HCT116 cells treated with 100 μM etoposide for the times indicated (*n* = 3). Nucleolar fusion is indicated. (H) Nucleolar area was measured with FIJI using manual segmentation of nucleoli from phase contrast images (*n* = 2, > 250 nuclei per treatment per experiment were analysed). Violin plots show pooled data. Statistical significance was calculated using Mann–Whitney (B) or Kruskal–Wallis test with Dunns multiple comparison (B, F, H). DAPI depicts DNA, molecular‐weight markers (kDa) are shown at left of immunoblots. Actin (sc‐47778) was used as a loading control. Scale bars are 10 μm throughout.

To investigate TIF‐IA's role in the SANP and senescence, we first examined levels of the protein after senescence induction. We utilised an established model of oncogene‐induced senescence (OIS) in which oestrogen receptor‐H‐RAS^G12V^ transduced IMR90 fibroblasts undergo a coordinated OIS response when exposed to 4‐hydroxytamoxifen (4‐OHT) (Figure S1a) (Innes and Gil 2019). ER:Stop cells, which proliferate normally in the presence of 4‐OHT, serve as controls. Based on prior studies with stress‐inducers, we expected TIF‐IA levels to decrease after senescence induction (Chen et al. 2018). However, 72 h following OIS induction we observed a significant *accumulation* of TIF‐IA, particularly in the nucleus and nucleolus (Figure 1A,B). To determine whether this unexpected response was time dependent, we performed kinetic studies and found that TIF‐IA accumulation was evident within 24 h of 4‐OHT exposure (Figure 1C,D and Figure S1b,c). Importantly, it paralleled a time dependent increase in nucleolar size, and reduction in nucleolar number (Figure 1D,E). It also preceded other key events reported in this model of OIS such as cell cycle arrest and increased β‐galactosidase activity (Innes 2018).

To test the broader relevance of these findings, we used a model of therapy‐induced senescence (TIS) in which cells are exposed to the DNA damaging agent, etoposide, for 48 h then recovered for 6 days to induce senescence (Figure S1d). Immunocytochemistry revealed TIF‐IA accumulation in both the nucleus and nucleolus as an early response to etoposide exposure (Figure 1F–H and Figure S1e,f). As with OIS, TIF‐IA accumulation was closely associated with nucleolar fusion, as indicated by nucleolar bridges and a marked increase in nucleolar size (Figure 1F–H and Figure S1e).

### 3.2 TIF‐IA Accumulates In Vivo, in Mouse Models of Senescence and Ageing

**FIGURE 2:**
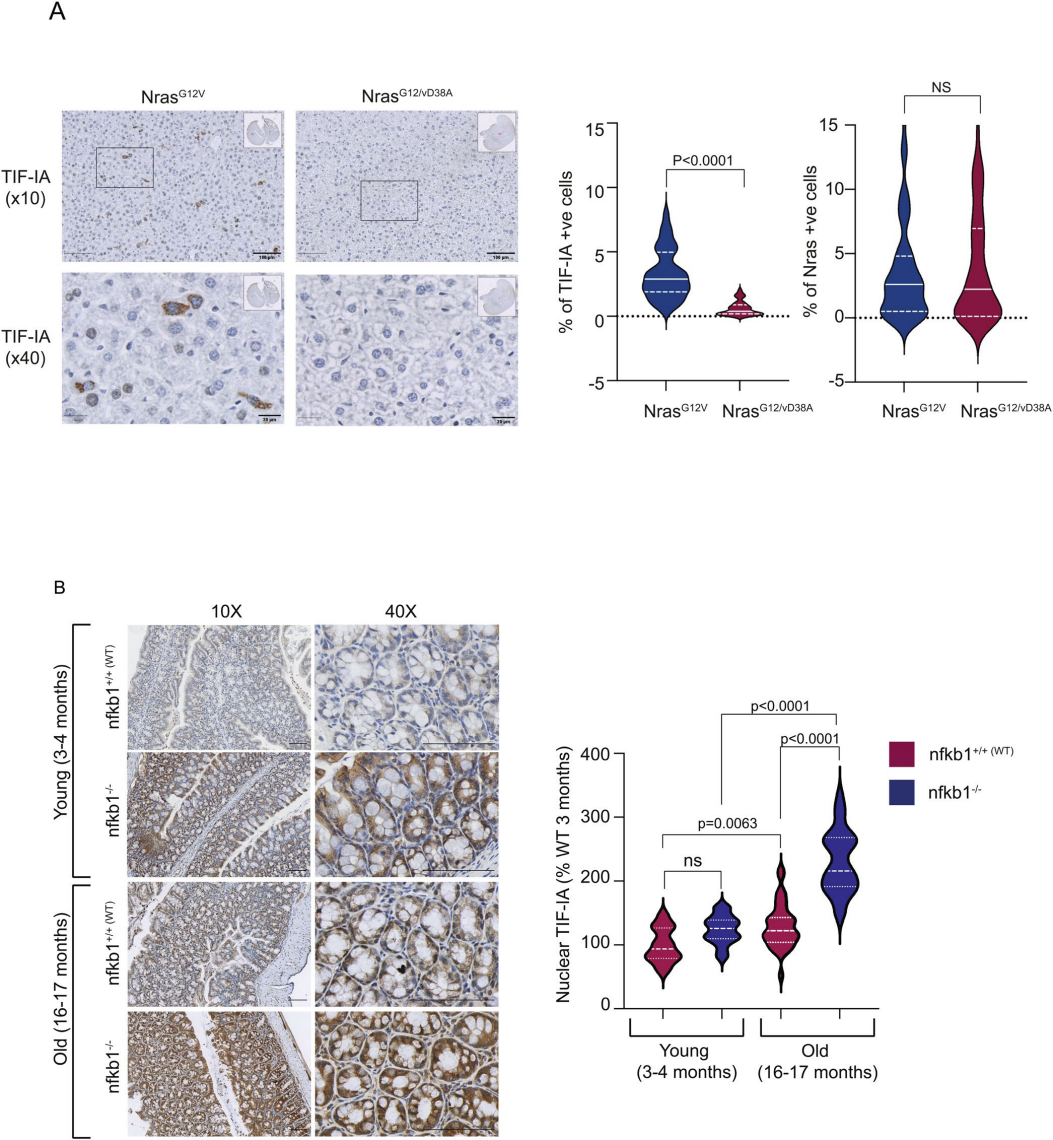
TIF‐IA protein accumulates in an animal model of OIS and in chronological and genetic models of ageing. (A) TIF‐IA and N‐ras immunohistochemistry were performed on liver sections from mice culled 6 days after hydrodynamic delivery of Nras^G12V/D38A^ (*n* = 5) or Nras^G12V^ (*n* = 5) transposons. Left: Representative images at 10× (scale bar 100 μm) and 40× (scale bar 20 μm) magnification. Right: QuPath software (v0.2.3) was used to quantify the percentage of TIF‐IA and N‐ras positive cells in five regions of interest (ROIs) per section. Pooled data (25 ROIs per condition) is shown. Mann–Whitney test determined significance. (B) Anti‐TIF‐IA immunohistochemistry was performed on colonic sections from young and old wild type (WT) and *nfkb1* ^*−/−*^ mice (*n* = 5 mice per condition). Left: Representative images at 10× and 40× magnification. Scale bar 20 μm. Right: QuPath software (v0.2.3) was used to quantify nuclear TIF‐IA intensity in five ROIs as above. Pooled data are shown. A Kruskal–Wallis test with Dunns multiple comparison was used to determine significance. ns, nonsignificant.

Next, we explored TIF‐IA accumulation in vivo using three well characterised mouse models of senescence and ageing. Firstly, we used a murine model in which OIS is induced in hepatocytes by expression of mutant Nras^G12V^ (Figure 2A). A plasmid encoding a mutant incapable of downstream Nras signalling (Nras^G12V/D38A^) was used as a negative control. Sections of livers from these mice were provided by the Acosta lab who previously demonstrated that 6 days post transduction, there is a significant increase in the expression of senescence markers dcr2, arf and Il‐1β in livers of mice expressing Nras^G12V^, compared to those expressing the control Nras^G12V/D38A^ (Hari et al. 2019). Here we found that 6 days after transduction there was a significant increase in TIF‐IA positive cells in livers of mice expressing the oncogenic Nras^G12V^, compared to those expressing the Nras^G12V/D38A^ control (Figure 2A). Furthermore, the relative increase in TIF‐IA positive cells upon Nras^G12V^ expression was consistent with the increase in expression of the above senescent markers (Hari et al. 2019). Nras staining showed that differences in TIF‐IA positivity were not due to varying Nras expression. It also confirmed that a comparable percentage of hepatocytes expressed Nras^G12V^ and TIF‐IA, further supporting a link (Figure 2A).

Next we used young (3–4 months) and older (16–17 months) wild type (WT) and *nfkb1* (p50) null (*nfkb1* ^*−/−*^) mice to explore TIF‐IA levels in chronological and genetically induced ageing. Up to 6 months, *nfkb1* ^*−/−*^ mice are comparable to their wild type counterparts (Bernal et al. 2014; Jurk et al. 2014). However, by 12 months they demonstrate distinctive, age‐related phenotypes including increased DNA damage, low‐grade inflammation, aggravated cell senescence and impaired regeneration in the gut (Yamini 2015). Quantitative immunohistochemistry in this model focussed on changes in nuclear TIF‐IA intensity, as this is the phenotype we observe in cells undergoing senescence. This revealed a significant increase in nuclear TIF‐IA in the intestines of older versus young mice for both groups (Figure 2B). In young mice, there was no significant difference in intestinal TIF‐IA levels between WT and *nfkb1* ^*−/−*^, in keeping with their similar phenotype at this age. However, in old mice, there was a highly significant increase in TIF‐IA in the *nfkb1* ^*−/−*^ group, compared to WT (Figure 2B).

Together, these findings demonstrate that TIF‐IA accumulation is a consistent response to senescence induction and ageing across various in vitro and in vivo models.

### 3.3 TIF‐IA Is Required for the Senescence‐Associated Nucleolar Phenotype

**FIGURE 3:**
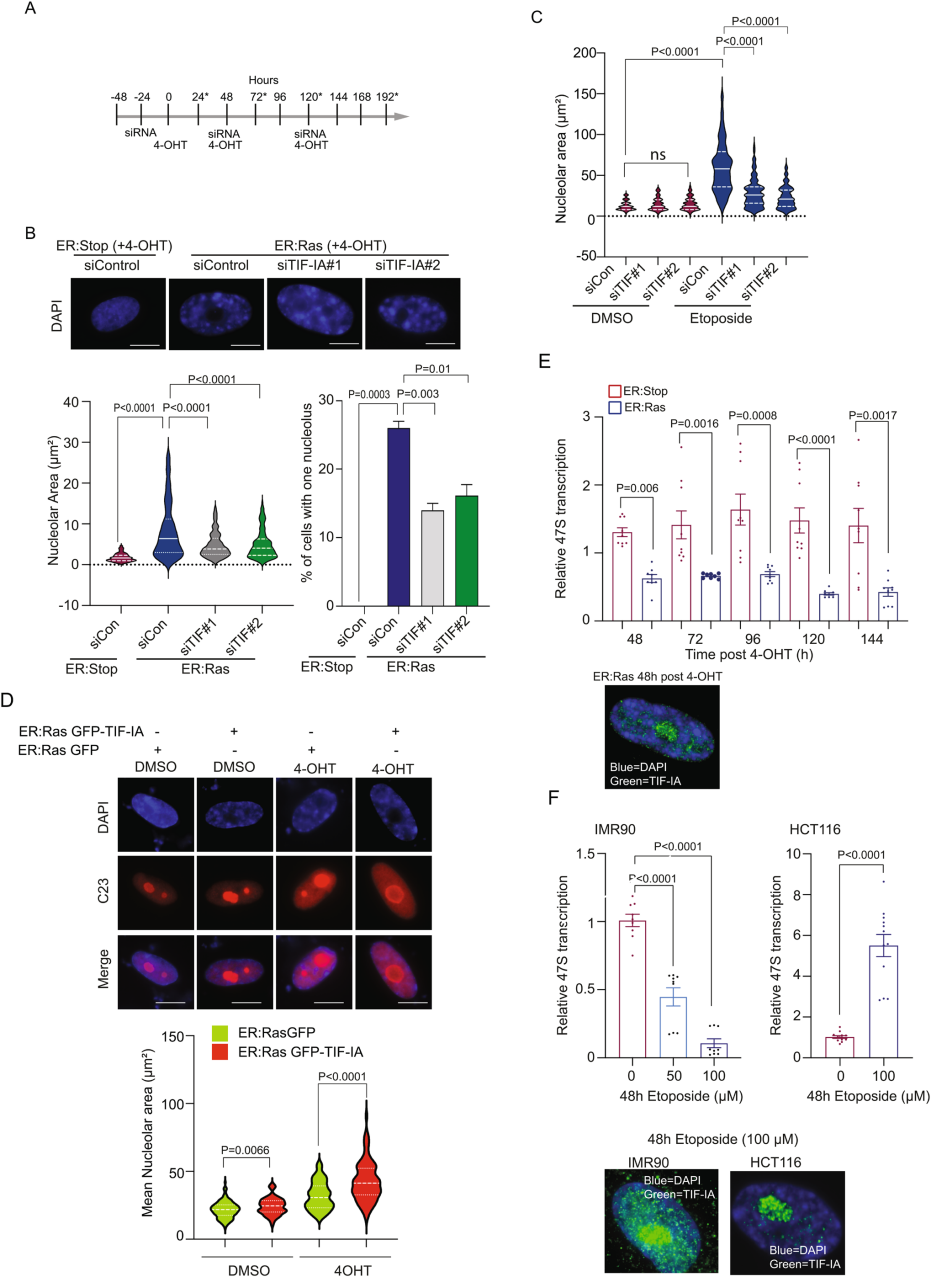
TIF‐IA depletion attenuates the senescence‐associated nucleolar phenotype in OIS and TIS. (A, B) IMR90 ER:Stop and ER: Ras fibroblasts were pretreated with two independent TIF‐IA siRNA (siTIF‐IA#1 and siTIF‐IA#2) or a nonsense sequence (siControl) prior to addition of 4‐OHT at various time points. (A) Schematic showing the treatment schedule. See Figure S2A for efficiency of TIF‐IA mRNA knockdown. (B) Top panel: Representative images of DAPI stained cells 48 h after 4‐OHT treatment (*n* = 2). Bottom panel left: nucleolar area was quantified as in Figure 1D. Pooled data for two experiments are shown. Bottom panel right: The percentage (±SE) of cells with a single, large nucleoli was determined manually using area devoid of DAPI staining. (C) HCT116 cells were transfected with control or two independent TIF‐IA siRNAs (siTIF‐IA #1, siTIF‐IA #2) prior to treatment with DMSO or 100 μM etoposide for 48 h. See Figure S2C for efficiency of TIF‐IA knockdown. Live images were acquired as in Figure 1G and nucleolar area determined using FIJI software. At least 125 nucleoli per treatment were assessed from three individual replicates. (D) IMR90 ER: Ras cells were transfected with pEGFP‐C1 or pEGFP‐TIF‐IA, treated for 5 days with DMSO or 4‐OHT then immunocytochemistry performed for the nucleolar marker, C23 (*n* = 3). Top: Representative immunomicrograph. Bottom: Nucleolar size was quantified using FIJI software segmenting on C23 stain. 33–100 cells were imaged per replicate. (E) TIF‐IA accumulation is associated with reduced 47S transcription in IMR90 cells. IMR90 ER: Stop and ER: Ras cells were exposed to 4‐OHT in time course experiments as in Figure 1C–E. qRT‐PCR was used to monitor 47S transcription. Each point represents a technical replicate (*n* = 3). Bottom: Immunomicrograph showing TIF‐IA nuclear/nucleolar accumulation 48 h after 4‐OHT exposure in ER: Ras cells. (F) qRT‐PCR was used to monitor 47S transcription in IMR90 (*n* = 3) and HCT116 (*n* = 4) cells treated with the given concentrations of etoposide for 48 h. Bottom: Representative immunomicrographs showing nucleolar accumulation of TIF‐IA. Statistical significance was determined using Kruskal–Wallis test with Dunns multiple comparison (B–D), one way ANOVA with Tukey's multiple comparison (B bottom right) or Student's *t*‐test (E, F). Scale bars are 10 μm throughout.

Next, we set out to definitively establish the role of TIF‐IA accumulation in the SANP. We used two independent siRNAs to deplete the protein before and during OIS initiation and qRT‐PCR to confirm efficient knockdown (Figure 3A and Figure S2a). We found that the increase in nucleolar area and reduction in nucleolar number observed upon OIS induction in control siRNA transfected cells was significantly abrogated when TIF‐IA was depleted (Figure 3B). In contrast to TIF‐IA, depleting PolI, which is also key for rDNA transcription, had no significant effect on nucleolar area after Ras^G12V^ induction (Figure S2b). Similar results were seen in the TIS model in that siRNA depletion of TIF‐IA abrogated the increase in nucleolar area observed after senescence induction (Figure 3C and Figure S2c). In contrast to nucleolar area, TIF‐IA depletion had no effect on nuclear area in OIS, indicating specificity (Figure S2d).

To further establish the link between TIF‐IA accumulation and the SANP, we overexpressed the protein in IMR90 fibroblasts. Using quantitative immunocytochemistry and C23 as a marker for nucleoli, we found that there was a significant increase in nucleolar area in cell populations transfected with GFP‐TIF‐IA compared to those transfected with GFP alone (Figure 3D). Furthermore, transfection with GFP‐TIF‐IA enhanced the effects of RAS^G12V^ induction on the nucleolar phenotype (Figure 3D).

Enlarged nucleoli are typically associated with enhanced ribosome biogenesis (Kasselimi et al. 2022). Given TIF‐IA's role in this process, its accumulation may be expected to increase rDNA transcription, causing nucleolar enlargement. Contrary to this expectation, qRT‐PCR analysis of the 47S pre‐RNA transcript revealed that compared to ER:Stop, there was a significant *reduction* in 47S rDNA transcription 24–72 h post 4‐OHT exposure in ER:Ras cells, despite evident TIF‐IA accumulation and nucleolar enlargement (Figure 1C,D) at these time points (Figure 3E). Similarly, etoposide treatment of IMR90 cells led to TIF‐IA accumulation and nucleolar enlargement, along with *decreased* 47S transcription (Figure 3F and Figure S1e,f). Conversely, etoposide treatment of HCT116 cells resulted in TIF‐IA accumulation, nucleolar enlargement, and *increased* 47S transcription (Figures 1F–H and 3F). As expected, siRNA depletion of both TIF‐IA and PolI significantly reduced 47S transcription in IMR90 cells (Figure S2e). However, only TIF‐IA depletion notably abrogated the effects of OIS induction on nucleolar area (Figure S2b).

Together, these data indicate that TIF‐IA accumulation is a pivotal regulator of the nucleolar phenotype in senescence and suggest that this is independent of nuclear size and 47S transcription.

### 3.4 TIF‐IA Is Not Required for Cell Cycle Arrest in Senescence, but Is Essential for the Expression of SASP Factors

**FIGURE 4:**
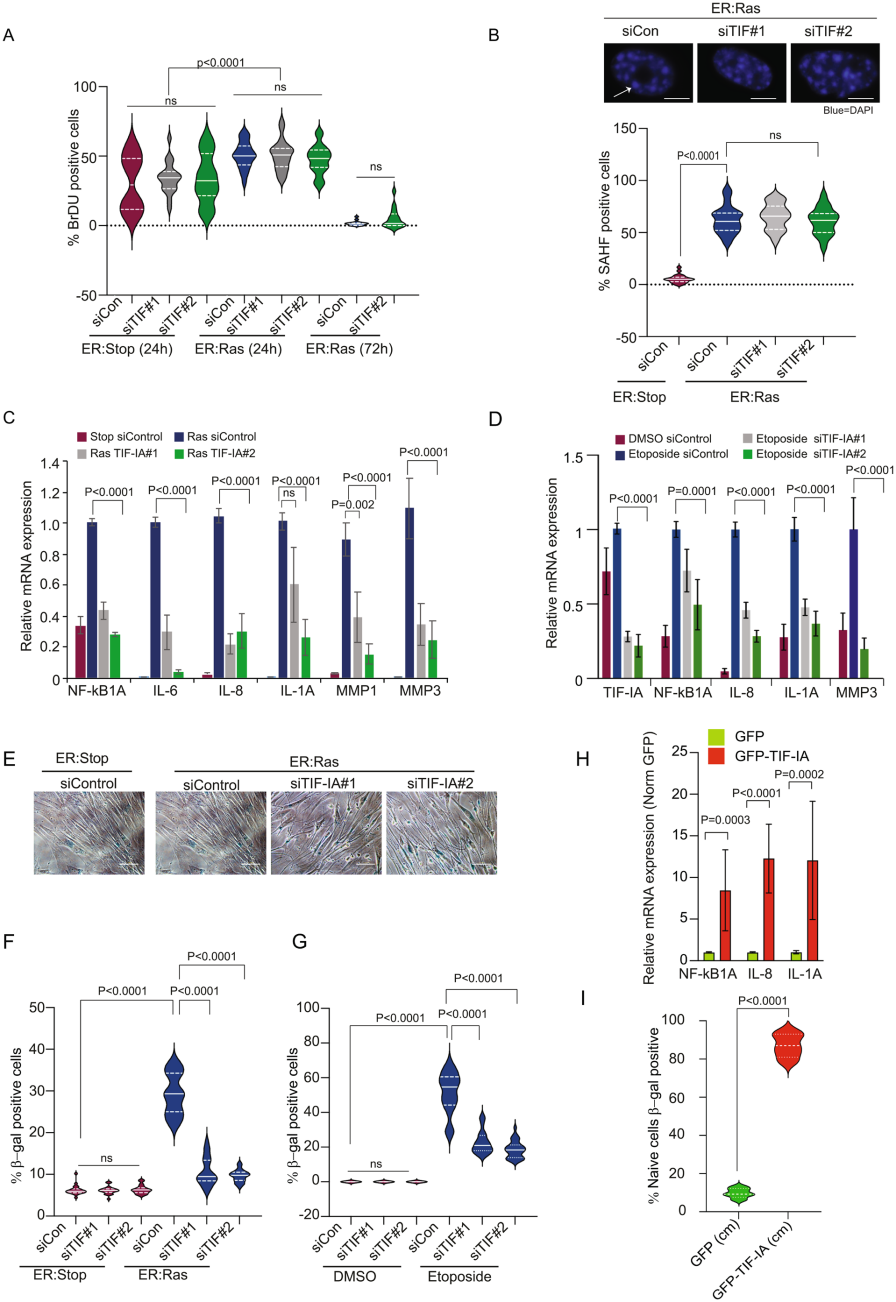
TIF‐IA depletion attenuates the transcription of SASP factors and the establishment of senescence in OIS and TIS, but is dispensable for cell cycle arrest. (A–C, F, G) IMR90 ER:Stop and ER:Ras fibroblasts were pretreated with two independent TIF‐IA siRNA (siTIF‐IA#1 & siTIF‐IA#2) or a nonsense sequence (siControl) prior to addition of 4‐OHT at various time points, as outlined in Figure 3A. (A) Anti‐BrdU immunocytochemistry, with manual quantification of positive cells, was used to assess cell proliferation. At least 100 cells from at least 10 fields of view were analysed per treatment group per experiment (*n* = 2). (B) The percentage of cells with senescence‐associated heterochromatin foci (SAHF) (large, bright DAPI foci indicated by the white arrow) was determined by microscopic analysis of the DAPI channel 120 h after 4‐OHT exposure. A minimum of 200 cells were counted manually per treatment for each replicate (*n* = 2). (C) qRT‐PCR demonstrating TIF‐IA depletion inhibits Ras‐induced transcription of SASP factors. All conditions for each gene are presented relative to ER:Ras siControl. Mean ± SEM (*n* = 3) is shown. (D) HCT116 cells were transfected with control or two independent TIF‐IA siRNAs (#siTIF‐IA 1, #siTIF‐IA 2) prior to treatment with 100 μM etoposide for 48 h, as outlined in Figure 3C. qRT‐PCR quantified gene expression. Bars represent the relative expression compared to etoposide siControl ± SEM (*n* = 3). (E, F) IMR90 cells were siRNA transfected and treated with 4‐OHT for 8 days as outlined above in Figure 3C. The effect of TIF‐IA knockdown on senescence was determined using β‐Galactosidase (β‐Gal) assays. (E) Typical fields of view after treatment. Blue indicates β‐Galactosidase positive cells. (F) The percentage of positive cells was quantified in 10 fields of view per replicate by light microscopy (*n* = 3). (G) HCT116 cells were transfected with control or TIF‐IA siRNA then treated with DMSO or etoposide as outlined above. β‐galactosidase assays were performed then percentage of senescent cells (indicated by blue stain) was determined by light microscopy. Ten fields of view, at least 50 cells/field, were counted for each experimental condition (*n* = 3). (H) HCT116 cells were transfected with pEGFP‐C1 or pEGFP‐TIF‐IA. qRT‐PCR was used to determine the levels of the given NF‐κB target genes 72 h later. GFP expression (relative to GAPDH) was used to normalise for transfection efficiency. (I) HCT116 cells were transfected with pEGFP‐C1 or pEGFP‐TIF‐IA. Forty‐eight hours later, conditioned media (cm) from transfected cells was transferred to naïve HCT116 cells. Following 8 days exposure to conditioned medium, β‐galactosidase assays were performed on the originally naïve cells as above. Ten fields of view, at least 50 cells/field, were counted for each experimental condition (*n* = 5). Pooled data for all repeats are shown throughout. Significance was determined by Kruskal–Wallis test with Dunns multiple comparison (A, B, F, G), one way ANOVA with Tukey's correction (C, D, H) or a Student's *t*‐test (I). ns, nonsignificant. Scale bars are 10 μm (B) or 100 μm (E).

Nucleolar changes in senescence have been linked to cell cycle arrest (Kasselimi et al. 2022). Therefore, we next used BrdU incorporation assays to explore the role of TIF‐IA accumulation in this endpoint. We found depletion of TIF‐IA (as outlined in Figure 3A) had no effect on the initial hyper‐proliferation observed after RAS^G12V^ induction, which is associated with DNA damage and full senescence, or on the subsequent cell cycle arrest (Figure 4A). Similarly, TIF‐IA depletion had no effect on the formation of senescence‐associated heterochromatin foci (SAHF), regions of condensed chromatin that form as a consequence of DNA damage (Figure 4B) (Narita et al. 2003).

Senescent cells secrete a mix of factors called the senescence‐associated secretory phenotype (SASP) which is essential for paracrine signalling, immunosurveillance and established senescence (Acosta et al. 2008; Kuilman et al. 2008). The NF‐κB transcription factor is a key regulator of the SASP but is dispensable for the cell cycle effects of senescence (Acosta et al. 2013). Given we previously linked TIF‐IA to NF‐κB in stress response (Chen et al. 2018) we next explored the relationship between TIF‐IA and SASP factor transcription in senescence.

Time‐course studies revealed TIF‐IA accumulation and nucleolar alterations precede SASP factor transcription in both OIS and TIS (Figure 1C–E and Figure S3a–e). TIF‐IA knockdown also significantly reduced the transcription of NF‐κB target genes and SASP factors following OIS and TIS induction in multiple cell types (Figure 4C,D and Figure S3f). Consistent with its effects on the SASP, TIF‐IA depletion impaired the establishment of senescence, as shown by altered cell morphology and reduced β‐galactosidase activity (Figure 4E–G). Conversely, GFP‐TIF‐IA overexpression enhanced SASP gene transcription compared to GFP control (Figure 4H). Furthermore, conditioned media from GFP‐TIF‐IA–overexpressing cells was sufficient to induce senescence in naïve HCT116 cells, as indicated by increased β‐galactosidase activity compared to GFP control, confirming secretion of functional SASP factors upon TIF‐IA protein accumulation (Figure 4I and Figure S3g).

Together our data indicate that TIF‐IA accumulation in nuclei and nucleoli is an early and key event in the SANP, the SASP and senescence induction.

### 3.5 TIF‐IA Binds to the Autophagy Receptor, p62, in Proliferating Cells

**FIGURE 5:**
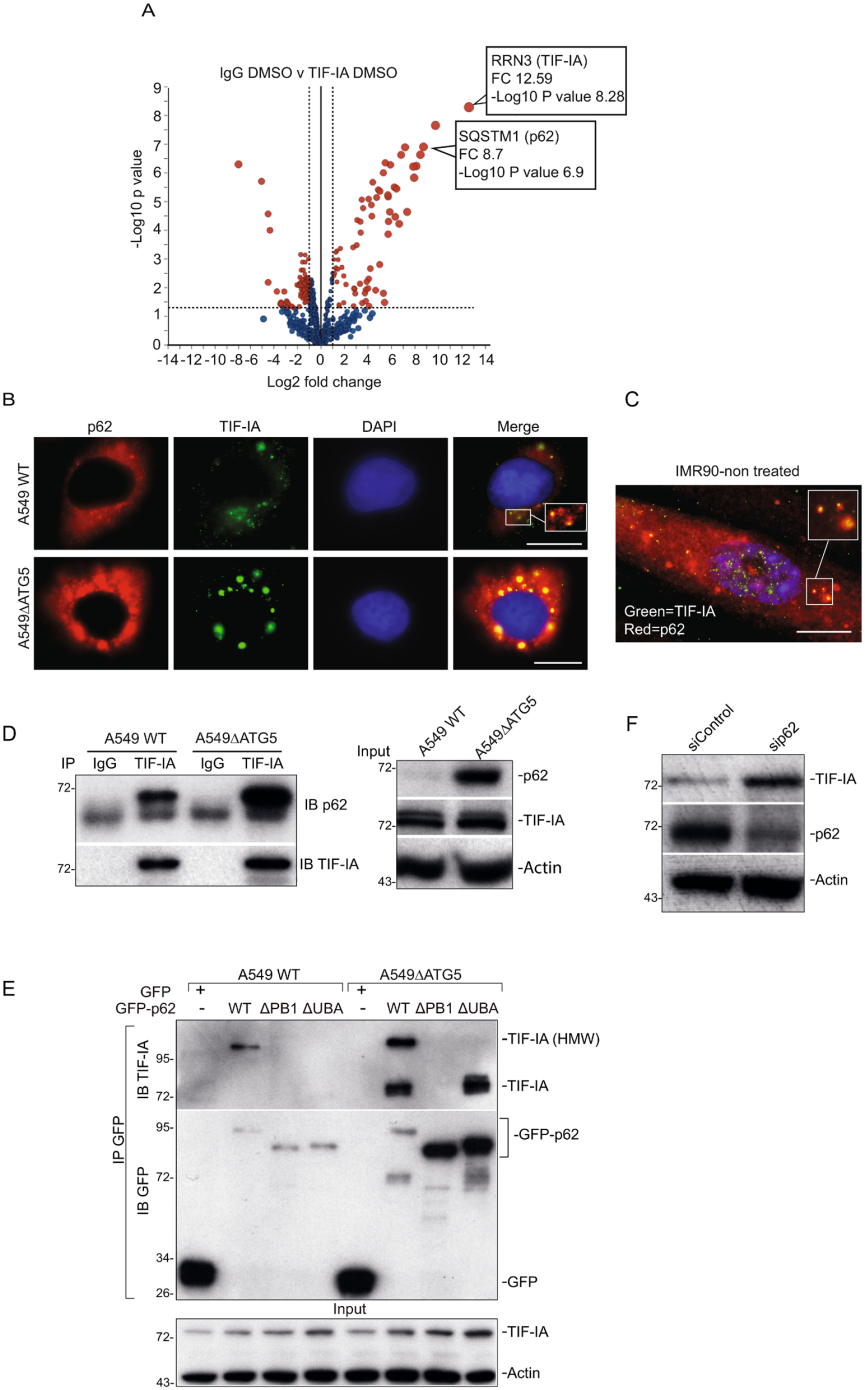
p62 binds to TIF‐IA and regulates levels of the protein in proliferating cells. (A) HCT116 cells were treated with DMSO (control) or etoposide (100 μM) for 8 h. Whole cell lysates were immunoprecipitated with mouse IgG or TIF‐IA antibody (Sc‐393460) then precipitated proteins analysed by quantitative mass spectrometry. Volcano plot shows a comparison of IgG versus TIF‐IA for DMSO treated cells. Blue dot = nonsignificant. Red dot = significant change (fold change [FC] > 2, *p* < 0.05). (B) Representative immunomicrographs (63×) showing level and localisation of TIF‐IA and p62 in wild type (WT) A549 cells and those in which the autophagy gene, *ATG5*, has been deleted (ΔATG5). Inset shows co‐localised foci in WT cells (*n* = 5). (C) Immmunomicrograph showing p62‐TIF‐IA co‐localisation in cytoplasmic foci in IMR90 cells. (D) Endogenous TIF‐IA was immunoprecipitated (IP) from WT or ΔATG A549 cells with rabbit TIF‐IA antibody (B8433) then recovered proteins analysed by immunoblot (IB) for p62 (BD610833). Stripped gels were re‐probed for mouse TIF‐IA (Sc‐393460). Input levels of proteins are shown. Rabbit IgG acts as a control (*n* = 3). (E) p62 binds to a high molecular weight (HMW) form of TIF‐IA in a manner dependent on the dimerization and ubiquitin binding domains. Wild type and ΔATG5 A549 cells were transfected with plasmids expressing GFP‐p62 WT, GFP‐p62ΔPB1 (deleted for the PB1 domain required for dimerization) or GFP‐p62ΔUBA (deleted for the ubiquitin binding domain). GFP‐tagged proteins were immunoprecipitated (IP) from whole cell lysates using GFP‐TRAP beads. Precipitated proteins were subjected to immunoblotting (IB) for TIF‐IA (Sc‐393460) and GFP (sc‐8433) (*n* = 2). Input levels of TIF‐IA are shown. (F) Immunoblot showing levels of TIF‐IA (sc‐8433) and p62 (BD610833) in whole cell lysates following siRNA depletion of p62 (*n* = 2). Molecular‐weight markers (kDa) are shown at left of immunoblots. Actin (sc‐47778) is used as a loading control throughout. Scale bars = 10 μm.

To further understand the role of TIF‐IA in the SANP and SASP, we used immunoprecipitation and quantitative mass spectrometry to identify novel TIF‐IA interactors that are potential effectors of this function. We chose the 8 h post etoposide timepoint as TIF‐IA accumulation and enlarged nucleoli were evident, but NF‐κB was not yet active (Figure S3b–e). The detailed results of the interactome studies are provided in Table S1. One of the top TIF‐IA interacting proteins was the autophagy cargo receptor, SQSTM1 (p62) (Figure 5A). This was of particular interest as p62 shuttles to the nuclei/nucleoli under stress (Lobb et al. 2021). It is also a regulator of the NF‐κB pathway and the SASP (Duran et al. 2008; Kang et al. 2015). Therefore, we further characterised this interaction.

Immunocytochemistry demonstrated TIF‐IA co‐localises with p62 in discrete cytoplasmic puncti in proliferating wild type A549 and IMR90 cells, and in large cytoplasmic complexes in A549 cells depleted for the autophagy component, ATG5 (ΔATG5) (Figure 5B,C). Immunoprecipitation followed by western blot analysis confirmed that TIF‐IA interacts with p62 in A549 cells, and that this interaction is enhanced by deletion of ATG5 (Figure 5D). Further mapping of the interaction in WT and ΔATG5 A549 cells revealed that wild type p62 binds to both a native and a high molecular weight (HMW) form of TIF‐IA (Figure 5E). Binding to both forms is lost upon deletion of the p62 PB1 domain (which is responsible for homodimerization and PKCα binding) while binding to HMW TIF‐IA is lost upon deletion of the ubiquitin binding domain (UBA) (Figure 5E). Based on these data, we hypothesise that p62 regulates basal levels of TIF‐IA by targeting a ubiquitinated form of the protein for degradation. In keeping with this suggestion, siRNA depletion of p62 causes a significant increase in TIF‐IA protein levels (Figure 5F).

### 3.6 p62‐TIF‐IA Binding Is Lost in Senescence in a Manner Dependent on the DNA Damage Response Kinase, ATM

**FIGURE 6:**
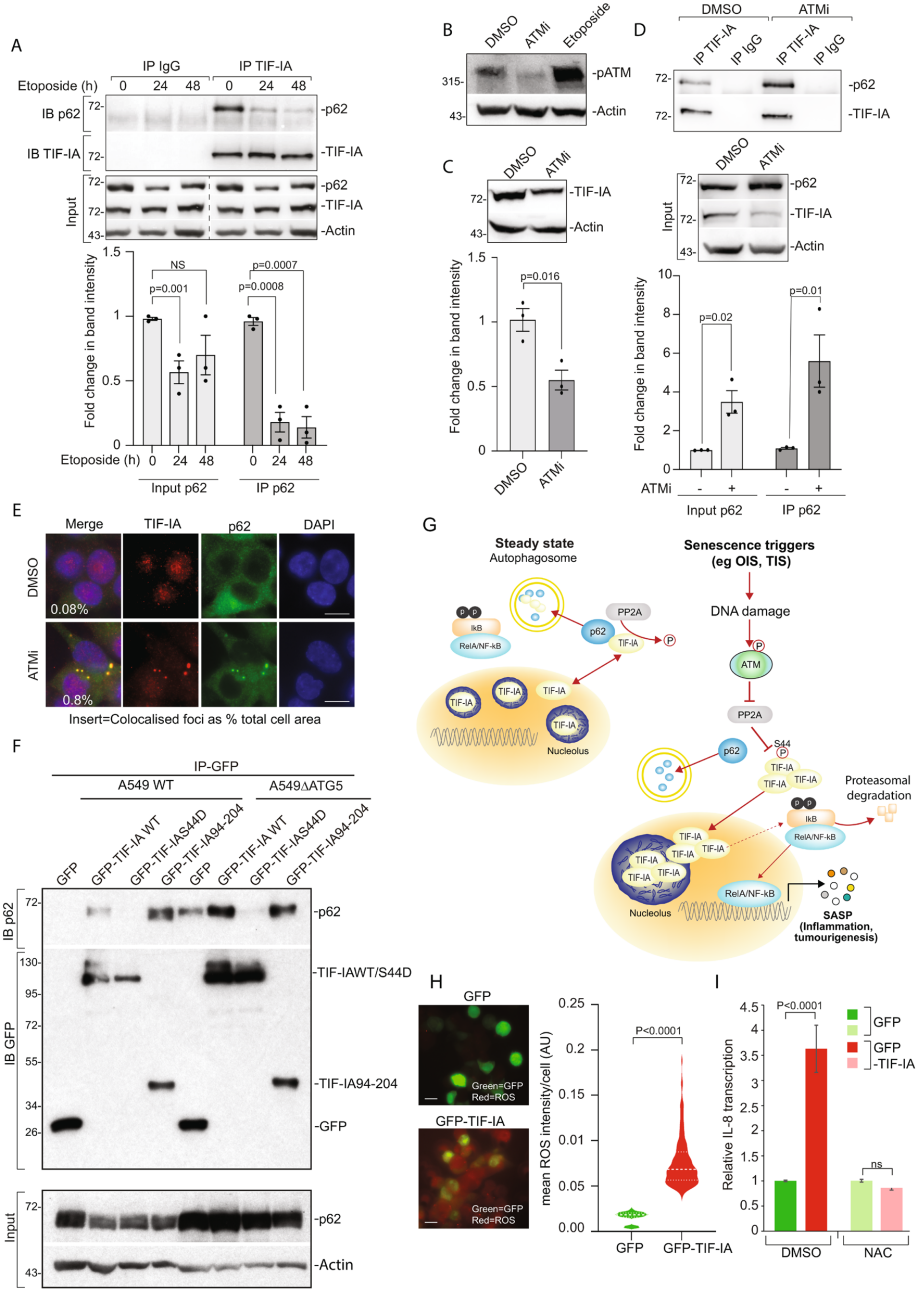
p62‐TIF‐IA binding is lost in senescence in a manner dependent on the DNA damage response kinase, ATM. (A) TIF‐IA was immunoprecipitated (IP) from HCT116 cells treated with etoposide (100 μM) for the indicated times then recovered proteins analysed by immunoblot (IB) for p62. Stripped gels were re‐probed for TIF‐IA. Input levels of protein are shown. Rabbit IgG acts as a control. p62 intensity was quantified by FIJI for input and IP. Mean ± SEM is shown (*n* = 3). (B) Anti‐phosphorylated ATM (pATM) (CST 5883) immunoblot performed on whole cell lysates from HCT116 cells treated with a small molecule ATM inhibitor (ATMi) (10 h), etoposide (24 h) or carrier control (DMSO). (C) HCT116 cells were treated with ATMi for 8 h then western blot analysis performed on whole cell lysates using the indicated antibodies. Top: Representative immunoblot. Bottom: Fiji was used to quantify TIF‐IA band intensity relative to Actin. Mean ± SEM is shown (*n* = 3). (D) HCT116 cells were treated with DMSO or ATMi for 8 h. TIF‐IA was immunoprecipitated and precipitates immunoblotted for p62 and TIF‐IA as in Figure 5. Representative immunoblots and input levels are shown. Rabbit IgG acts as a control. Below: Fiji was used to quantify band intensities as in A. Mean ± SEM is shown (*n* = 3). (E) Representative immunomicrographs demonstrating level and localisation of TIF‐IA in HCT116 cells treated with carrier (DMSO) or ATMi. FIJI was used to quantify the percentage of cell area occupied by yellow foci (*n* = 2). (F) Wild type and ΔATG5 A549 cells were transfected with plasmids expressing GFP, GFP‐TIF‐IA WT, GFP‐TIF‐IA S44D (phospho mimetic) or GFP TIF‐IA 94–204. GFP‐tagged proteins were immunoprecipitated (IP) from whole cell lysates using GFP‐TRAP beads. Precipitated proteins were subjected to immunoblotting (IB) for p62 (BD610833) and GFP (sc‐8433) (*n* = 3). Input levels of p62 are shown. (G) Model of suggested TIF‐IA senescence pathway. In steady state, TIF‐IA shuttles between the nucleolus, nucleoplasm and cytoplasm. PP2A dephosphorylates TIF‐IA at S44 allowing p62 binding and degradation. Upon senescence induction, DNA damage activates ATM which inhibits PP2A activity and thus, TIF‐IA S44 dephosphorylation and p62 binding. TIF‐IA consequently accumulates which causes nucleolar fusion, NF‐κB pathway activation and the SASP. See discussion for further details. (H) HCT116 cells were transfected with pEGFP‐C1 or pEGFP‐TIF‐IA as in Figure 4. Twenty‐four hours later, CellRox Deep Red Reagent was used to identify reactive oxygen species (ROS) and Cell Profiler used to quantify intensity. Left: Representative immunomicrographs. Right: Violin plots show pooled mean cytoplasmic intensity/cell for three experiments. At least 330 cells (independent of GFP positivity) were analysed per condition. (I) HCT116 cells were pretreated for 24 h with the ROS scavenger, NAC, prior to 48 h transfection with pEGFP‐C1 or pEGFP‐TIF‐IA in the presence of NAC. qRT‐PCR quantified IL‐8 expression. All bars are relative to EGFP treated with DMSO. Molecular‐weight markers (kDa) are shown at left of immunoblots. Actin acts as a loading control throughout. Scale bar = 10 μm. Significance was determined using a Student's *t*‐test (A, C, D), a Mann–Whitney test or one way ANOVA with Tukey's multiple comparison (I). ns, nonsignificant.

Next, we explored the effects of senescence induction on the TIF‐IA‐p62 interaction. Immunoprecipitation followed by immunoblot analysis revealed a significant reduction in p62‐TIF‐IA binding in response to TIS (Figure 6A). Similar results were also observed after induction of OIS (Figure S4a). Quantification of immunoblots showed that p62 levels declined after etoposide exposure; however, the reduction in p62‐TIF‐IA binding was more pronounced (Figure 6A and Figure S4a). This suggests that the reduced binding is not just a consequence of p62 degradation, but that a specific pathway may disrupt the interaction after senescence induction.

DNA damage is a common feature of both OIS and TIS and a key contributor to senescence and ageing (Schumacher et al. 2021). Depletion of TIF‐IA did not affect the hyper‐proliferative stage of OIS, during which DNA damage occurs, or the formation of SAHF, which is linked to DNA damage (Figure 4A,B). Therefore, we hypothesised that the p62‐TIF‐IA interaction is lost downstream of DNA damage, leading to TIF‐IA accumulation.

Ataxia‐telangiectasia mutated kinase (ATM) is a DNA damage response (DDR) kinase that is activated in the early stages of OIS and TIS, and that plays a particularly important role in driving the SASP, senescence and ageing (Figure 6B) (Qian et al. 2018; Zhao et al. 2020). Therefore, we explored the role of the kinase in the TIF‐IA‐p62 interaction. Immunoblot and immunoprecipitation assays demonstrated that inhibition of ATM activity, using the ATM inhibitor KU55933 (ATMi), caused a significant reduction in TIF‐IA protein and an increase in TIF‐IA‐p62 binding (Figure 6B–D). Additionally, there was an increase in TIF‐IA‐p62 co‐localised foci in the presence of the inhibitor (Figure 6E).

Protein phosphatase 2A (PP2A), which dephosphorylates serine 44 of TIF‐IA, is a direct target of the ATM kinase and its activity is inhibited upon ATM‐mediated phosphorylation (Li et al. 2012; Mayer et al. 2004). We had previously demonstrated that TIF‐IA accumulates when cells are exposed to the PP2A inhibitor, okadaic acid (Chen et al. 2018). We also demonstrated that mutating S44 of TIF‐IA to the phospho‐mimetic, aspartic acid (S44D), completely blocks stress‐induced degradation of the protein (Chen et al. 2018). Based on these data, we considered that S44 dephosphorylation is required for TIF‐IA to bind to p62 and that, by targeting PP2A, ATM blocks this dephosphorylation. Indeed, immunoprecipitation assays demonstrated that the TIF‐IA S44D mutant, which cannot be dephosphorylated, is unable to bind p62 (Figure 6F). Further deletion studies revealed that amino acid residues 94–204 of TIF‐IA are sufficient for the p62 interaction (Figure 6F).

Based on the above and previous data, we propose a model whereby in proliferating cells, TIF‐IA is targeted for degradation through its association with p62 (Figure 6G). However, during the early phases of senescence induction, ATM‐mediated inhibition of PP2A causes an accumulation of TIF‐IA phosphorylated at S44, loss of p62 binding, translocation of the protein to the nucleus/nucleolus and consequently, the SANP and SASP. In support of this model, we found that blocking TIF‐IA accumulation in TIS, using ATMi, significantly abrogated the nucleolar enlargement observed in response to etoposide (Figure S4b).

### 3.7 Increase in Reactive Oxygen Species (ROS) in Response to TIF‐IA Accumulation

Impairment of mitochondrial ribosome function causes ROS generation (Correia‐Melo et al. 2016; Davalli et al. 2016), which plays a key role in the SASP (Vizioli et al. 2020) and mimics the effects of senescence induction on nucleolar morphology (Jo et al. 2024). Given the TIF‐IA interactome was enriched for mitochondrial ribosomal proteins and included the key regulator of oxidative stress, KEAP‐1 (Table S1), we considered that TIF‐IA accumulation may act by causing oxidative stress. Indeed, CellRox deep red assays indicated that expression of GFP‐TIF‐IA caused a significant increase in ROS, compared to expression of GFP alone. Furthermore, the increase in *IL‐8* transcription observed upon GFP‐TIF‐IA expression was abrogated in the presence of the ROS scavenger, NAC (*N*‐acetyl‐l‐cysteine). Together, these data provide intriguing mechanistic insight into how TIF‐IA accumulation may cause the SASP and the SANP.

## 4 Discussion

Here we make the novel observations that TIF‐IA accumulates in senescence and in ageing tissue, that this accumulation is regulated by p62 downstream of ATM activation, and that TIF‐IA accumulation is causally involved in the senescence‐associated nucleolar phenotype (SANP), the senescence‐associated secretory phenotype (SASP) and established senescence. Our findings on a link between TIF‐IA accumulation and senescence are supported by Nishimura et al. (2015), who used overexpression of TIF‐IA to manipulate ribosome biogenesis. Like us, they found that this overexpression caused an increase in nucleolar area, secretion of SASP factors and senescence. However, unlike our study, Nishimura did not investigate the regulation of endogenous TIF‐IA following senescence induction. Thus, their work supports but does not detract from the novelty of our findings. Together, these data unveil a new signalling axis that has relevance to the chronic inflammatory phenotype observed in senescence and ageing.

The mechanism(s) by which TIF‐IA contributes to the reduction in nucleolar number and increase in nucleolar size in senescence remains unclear. Traditionally, increased nucleolar size was considered to be a direct consequence of increased rDNA transcription (Boulon et al. 2010). Indeed, several studies indicate enhanced ribosome biogenesis causes nucleolar morphological changes in senescence and ageing (Kasselimi et al. 2022; Lessard et al. 2018; Nishimura et al. 2015; Ren et al. 2019). However, our data suggest that TIF‐IA alters nucleolar morphology independent of effects on rDNA transcription. Across all models tested, TIF‐IA accumulation was causally associated with a reduction in nucleolar number and an increase in nucleolar size; yet, these changes showed no relationship with 47S rDNA transcription. In OIS, TIF‐IA accumulation and nucleolar enlargement were associated with a decrease in rDNA transcription at all timepoints, while in TIS, the impact varied by cell line. More recent data suggest that nucleolar remodelling in senescence may result from rapid coalescence of nucleolar droplets, rather than increased ribosome biogenesis (Caragine et al. 2018, 2019; Dillinger et al. 2017). Such fusion events are reported to either decrease or minimally affect 47S transcription (Caragine et al. 2018; Pundel et al. 2022). Consistent with this disconnect between nucleolar fusion and rDNA transcription, we and others have shown reduced nucleolar number coupled with increased nucleolar size in response to specific stress conditions, which are associated with reduced rDNA transcription (Chen et al. 2018; Fatyol and Grummt 2008). Here we knocked down PolI, which reduced 47S transcription but had no effect on nucleolar size. Together, these data support a model in which TIF‐IA promotes nucleolar coalescence in senescence, independent of its canonical role in ribosome biogenesis.

We show that TIF‐IA overexpression increases ROS levels, which may contribute to these nucleolar changes. In support of this idea, Jo et al. (2024) recently reported that the reactive oxygen species H_2_O_2_ reduces nucleolar number and enlarges nucleoli to a similar extent as etoposide, while Sapio et al. (2021) demonstrated that altered nucleolar redox state decreases 47S transcription and affects pre‐rRNA processing. Other mechanisms reported to regulate nucleolar number and size include modulation of chromatin dynamics, depletion of the nuclear membrane LINC (linker of nucleoskeleton and cytoskeleton) complex and damage to rDNA (Korsholm et al. 2019, 2020; Satomi et al. 2020). Interestingly, Pundel et al. (2022) recently demonstrated nucleolar fusion in response to changes in extracellular adhesive cues. This is particularly relevant to our study as two of the 19 proteins we found to specifically interact with TIF‐IA after etoposide treatment, KRT77 and VASP, are involved in signalling from the extracellular matrix (ECM) (Table S1). These data suggest a possible link between the ECM, TIF‐IA, ROS and nucleolar fusion, which warrants further investigation.

Oxidative stress is a hallmark of senescence that has been linked not only to changes in nucleolar structure, but also to the SASP (Correia‐Melo et al. 2016; Davalli et al. 2016; Nelson et al. 2018). Consistent with a causal role for ROS in TIF‐IA‐induced SASP, the increase we observed in *IL‐8* transcription following TIF‐IA overexpression was abrogated by the ROS scavenger NAC. The mechanisms through which TIF‐IA elevates ROS, and how this causes the SASP, remain to be defined. Vizioli et al. (2020) demonstrated that following senescence induction, mitochondrial ROS can activate JNK, leading to chromatin leakage and subsequent activation of the cGAS–STING–NF‐κB axis that drives the SASP. Understanding whether TIF‐IA acts within this pathway upstream of JNK, or whether its effects on nucleolar architecture indirectly promote chromatin release and NF‐κB activation, is now a priority. Elucidating this mechanism may uncover new strategies to suppress the SASP and mitigate age‐related pathologies.

We previously demonstrated that stress‐induced TIF‐IA *degradation* leads to reduced nucleolar number, increased nucleolar size, activation of the NF‐κB pathway, and apoptosis (Chen et al. 2018; Chen and Stark 2018). The data presented here, showing that TIF‐IA *accumulation* drives the SANP and SASP, would appear to contradict these findings. However, as both conditions involve loss of cytoplasmic TIF‐IA, either through nuclear translocation or degradation, it may be this loss that triggers downstream signalling to NF‐κB and the nucleolus. Alternatively, TIF‐IA may function as a ‘Goldilocks protein’, whereby both its accumulation and depletion disrupt similar signalling networks. Regardless of the precise downstream mechanisms by which TIF‐IA exerts its effects, together our data provide compelling evidence that TIF‐IA protein stability is a critical determinant of cellular homeostasis.

In this study, we identify a novel role for p62 in regulating this stability. Our data indicate that p62 binds a ubiquitinated form of TIF‐IA, and that dephosphorylation at S44 is required for this interaction. Our previous studies demonstrated that dephosphorylation of S44 is also required for stress‐mediated TIF‐IA degradation (Chen et al. 2018). This suggests a model in which stress or senescence signals regulate TIF‐IA turnover by altering the phosphorylation status of S44 and consequently, p62 binding and autophagic degradation. Previous studies demonstrated that TIF‐IA stability is regulated by MDM2‐mediated ubiquitination (Nguyen le and Mitchell 2013). Whether MDM2 facilitates p62–TIF‐IA binding, or acts in parallel to regulate distinct ubiquitin linkages, remains to be determined. Elucidating the upstream signalling networks that coordinate TIF‐IA ubiquitination, phosphorylation and p62 recruitment may uncover new targets to modulate inflammation and age‐related disease.

Senescent cells are detected at low levels in human tissues. For example, Guo et al. (2019) found that only 0.3% of cells in normal colonic mucosa express senescent markers. In our study, however, we observed *global* increases in TIF‐IA protein in colonic tissue from both chronologically and genetically (*nfkb1* ^*−/−*^) aged mice. Chronological ageing is characterised by widespread DNA damage, caused by genotoxic stress and reactive oxygen species, while in *nfkb1* ^*−/−*^ mice, chronic inflammation leads to telomere shortening and DDR activation (Jurk et al. 2014). Since we show that TIF‐IA accumulates downstream of DDR, the global increase we observe may represent a tissue‐wide response to genetic instability. Genetic instability can be a precursor to senescence but not all damaged cells will progress to a stable senescent state, especially at any given time. Thus, this may explain this difference in TIF‐IA levels and the percentage of senescent cells. Further studies are focussed on the use of higher order models to understand the relationship between DNA damage, TIF‐IA, senescence and ageing in vivo.

Understanding of nucleolar fusion in senescence and ageing is an emerging field, but remains enigmatic. The findings presented here, demonstrating TIF‐IA accumulates in senescence and ageing tissue, that this accumulation is regulated by p62, and that it is responsible for nucleolar fusion linked to transcription of inflammatory factors, are a major step forward. The data open up many new avenues of research in the ageing field.

## Author Contributions

L.A.S. supervised the project, designed the study, analysed the data and wrote the manuscript. H.C.T. designed the study, performed the experiments, analysed the data and prepared the manuscript. T.S.B., K.D., Y.Y., J.D., H.W., F.D., O.F.A., F.A., A.N., P.M., A.G., F.R.M. and A.V.K. performed the experiments and analysed the data. F.O. and J.C.A. supervised studies on murine models. All authors have reviewed and approved the manuscript.

## Funding

The work was funded by BBSRC (BB/S018530/1), Worldwide Cancer Research (25‐0415) and Rosetrees Trust (A631, JS16/M225) to L.A.S. and CRUK (C18342/A23390) to F.O and D.M.

## Conflicts of Interest

The authors declare no conflicts of interest.

## Supporting information


**Data S1:** acel70334‐sup‐0001‐DataS1.zip.

## Data Availability

The data underlying this article will be shared on reasonable request to the corresponding author.
