# Supplementary material for: Loss of p62 Binding Allows TIF‐IA Accumulation in Senescence, Which Promotes Phenotypic Changes to Nucleoli and the Senescence Associated Secretory Phenotype
Source: Aging Cell. 2025 Dec 29;25(1):e70334. doi: 10.1111/acel.70334 (PMC12748510; doi:10.1111/acel.70334)
Supplement: Supplementary file 1 — Data S1: acel70334‐sup‐0001‐DataS1.zip. [file ACEL-25-e70334-s001.zip › acel70334-sup-0003-FigureS2@Supplemental figure legends_revised2.docx]

**Supplemental figure legends**

**Supplemental Figure 1.** (A) Schematic outlining the model of oncogene-induced senescence (OIS) used. ER:RAS cells treated with 4-hydroxytamoxifen (4-OHT) undergo senescence while ER:STOP cells continue to proliferate. (B and C) IMR90 ER:STOP or ER:RAS cells were treated with 4OHT in time course studies. (B) Anti-TIF-IA immunoblots were performed on whole cell lysates. FIJJI was used to quantify TIF-IA band intensity relative to actin (see Figure 1c for a representative image). N=3. (C) Anti-TIF-IA immunocytochemistry was performed. Representative immunomicrohraphs are shown. Image quantification of TIF-IA intensity, nucleolar area and nucleolar number can be found in Figures 1d and e. N=5. (D) Schematic outlining the model of therapy-induced senescence (TIS) used. (E and F) IMR90 cells were treated with DMSO or etoposide for the time specified. (E) Left: Immunomicrographs demonstrating the localisation and intensity of TIF-IA. Right: FIJJI was used to measure nuclear TIF-IA intensity. Five fields of view per experiment (at least 150 cells) were captured. N=5. (F) Representative immunoblot showing whole cell levels of TIF-IA (n=2). (G) A549 and HCT116 cells were treated with DMSO (0) or etoposide (100uM) for 48h then anti-TIF-IA immunocytochemistry performed. Left: Representative immunomicrographs. Right: Cell profiler was used to quantify nuclear intensity of TIF-IA in at least 5 fields of view (minimum 200 cells) per experiment. N=3. Students Ttest (B) or Kruscal-Wallis test with Dunns multiple comparison (E and G) were used to determine significance. Scale bars=10 µm. Molecular-weight markers (KDa) are shown at left of immunoblots

**Supplemental Figure 2**. (A and D) IMR90 ER:Stop and ER:Ras fibroblasts were pre-treated with a non-sense control siRNA (SiCon) or two independent TIF-IA siRNAs (SiTIF-IA#1 & SiTIF-IA#2) prior to addition of 4-OHT. See figure 3a for schematic. (A) qRT-PCR was used to monitor TIF-IA expression relative to GAPDH. (D) FIJI™ software was used to quantify nuclear area (depicted by DAPI stain). Pooled data for 2 experiments is shown (minimum 111 cells per group). (B) IMR90 ER:Ras fibroblasts were pre-treated with control siRNA (SiCon), TIF-IA siRNA (SiTIF-IA#2) or PolI siRNA (siPolI) prior to addition of 4-OHT. Cells were fixed, DAPI stained and nucleolar area quantified as outlined in Figure 3B (n=2, minimum 78 cells per condition). (C) HCT116 cells were transfected with control or two independent TIF-IA siRNAs (#siTIF-IA 1, #siTIF-IA 2) prior to treatment with DMSO (control) or 100uM Etoposide for 48h. qRT-PCR was used to monitor TIF-IA expression relative to GAPDH. Mean +/- SE is shown (n=3). (E) IMR90 ER:RAS fibroblasts were transfected with siRNA prior to 4-OHT treatment as in B. qRT-PCR was used to monitor 47S transcription (n=2). One way Anova with Tukey’s correction (A, D, E) or Kruscal-Wallis test with Dunns multiple comparison (B, C), were used to determine significance.

**Supplemental Figure 3** (A) IMR90 ER:Stop and ER:Ras fibroblasts were treated with 4-OHT for the times indicated. qRT-PCR was used to monitor expression of the early NF-κB target gene, *NFKB1A* (IκB), relative to GAPDH. Mean +/- SE is shown. N=3 (B, C and E) HCT116 cells were treated with DMSO (0h) or etoposide (100uM) for the times shown. (B and C) Anti-TIF-IA immunocytochemistry was performed. (B) Representative immunomicrographs demonstrate TIF-IA localisation. (C) FIJI™ software was used to quantify nucleolar area (depicted by areas devoid of DAPI stain). Kruscal-Wallis test with Dunns multiple comparison was used to determine significance. (E) qRT-PCR was used to monitor expression of the NF-κB target and SASP factor, IL-8. P values derived using a student T test. (D) HCT116 cells were transfected with the 3enhancer ConA-Luc NF-κB reporter plasmid and the pCMV-βgal control plasmid prior to etoposide treatment for the times specified. Mean (+/- SE) NF-κB activity, relative to β-gal, is shown. N=3. (F) A549 and HCT116 cells were pre-treated with control siRNA (SiCon) or TIF-IA#2 siRNA prior to 48h treatment with DMSO or Etoposide (100uM). qRT-PCR was used to monitor expression of TIF-IA and the SASP factors shown. Mean relative mRNA expression +/- SE is shown. N=3. P values derived using one way Anova with Tukey’s correction. (G) HCT116 cells were transfected with pEGFP-C1 or pEGFP-TIF-IA. Forty-eight hours later, conditioned media (cm) from transfected cells was transferred to naïve HCT116 cells. Following 8 days exposure to conditioned medium, β-galactosidase assays were performed on the originally naïve cells. Representative images are shown. Scale bars=10 µm (B) and 50 µm (G).

**Supplemental Figure 4** ER:RAS cell were treated with DMSO or 4-OHT for 72h. TIF-IA was immunoprecipitated (IP) then recovered proteins analysed by immunoblot (IB) for p62. Stripped gels were re-probed for TIF-IA. Input levels of protein are shown. Rabbit IgG acts as a control. p62 intensity was quantified by ImageJ for input and IP. N=2. Mean +/- SEM is shown. P value derived using a student Ttest. Molecular-weight markers (KDa) are shown at left of immunoblots
