# Supplementary figures and images for: Loss of p62 Binding Allows TIF‐IA Accumulation in Senescence, Which Promotes Phenotypic Changes to Nucleoli and the Senescence Associated Secretory Phenotype
Source: Aging Cell. 2025 Dec 29;25(1):e70334. doi: 10.1111/acel.70334 (PMC12748510; doi:10.1111/acel.70334)

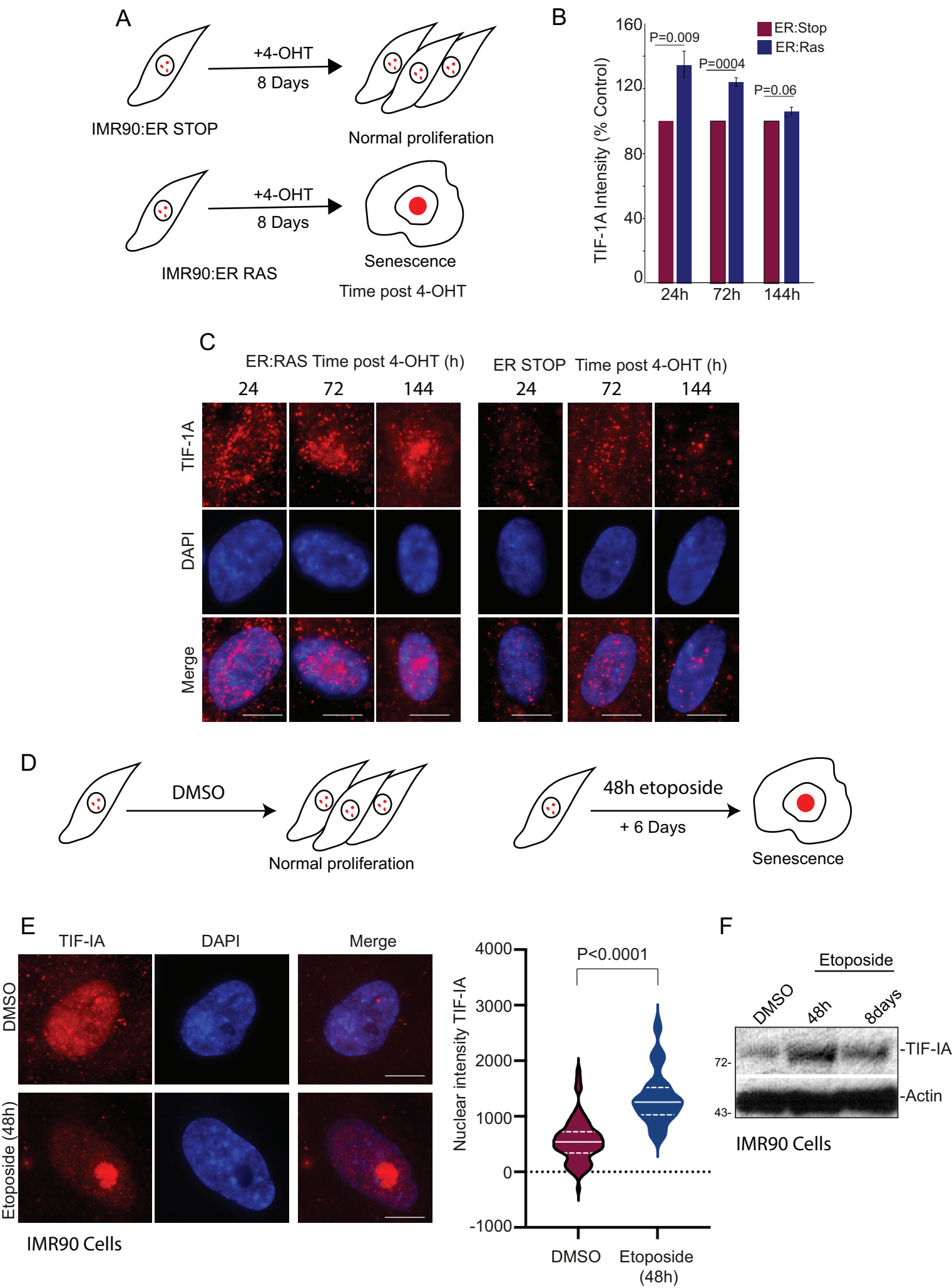

Supplement: Supplementary file 1 — Data S1: acel70334‐sup‐0001‐DataS1.zip. [file ACEL-25-e70334-s001.zip › acel70334-sup-0001-FigureS1.pdf]

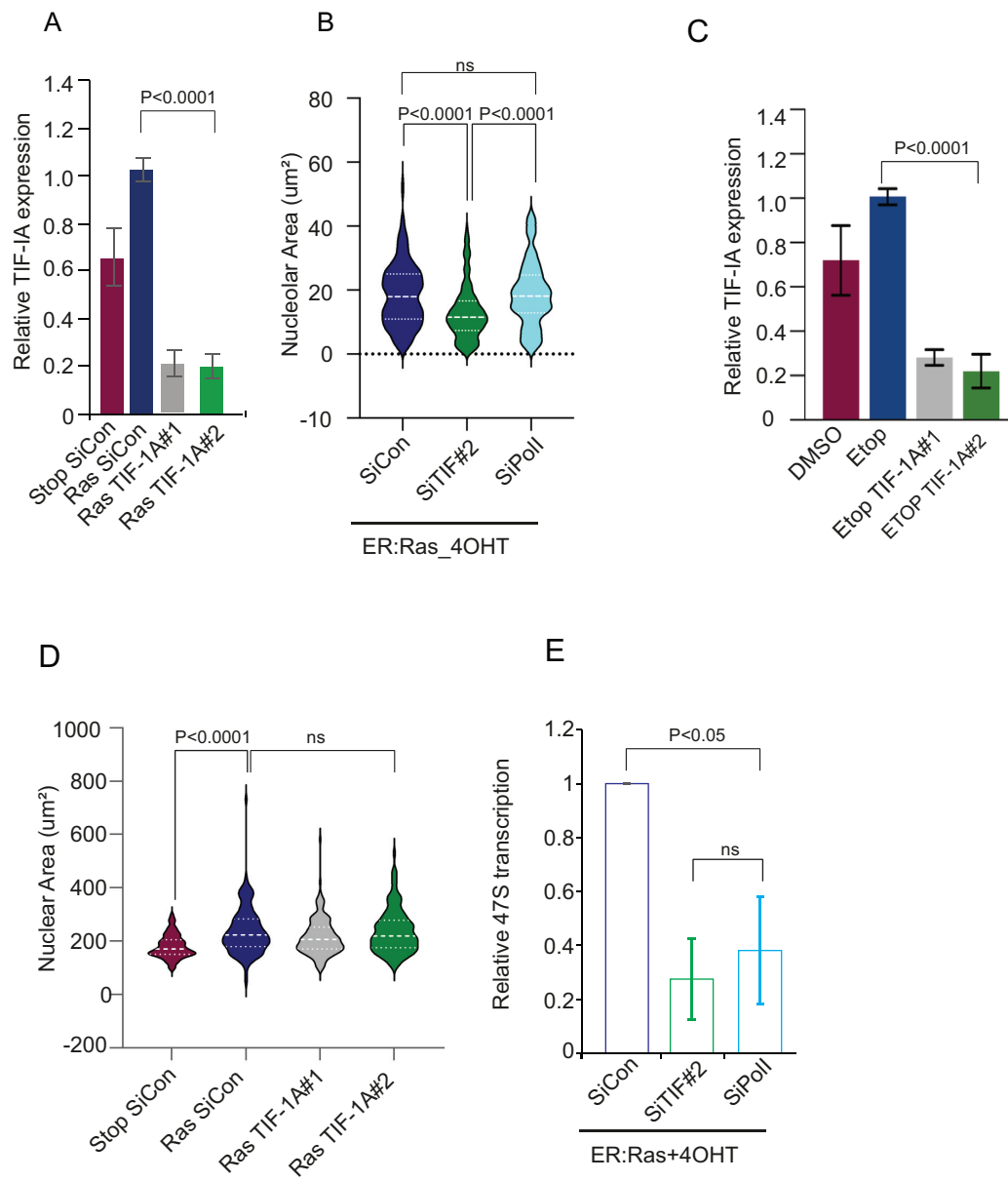

Supplement: Supplementary file 1 — Data S1: acel70334‐sup‐0001‐DataS1.zip. [file ACEL-25-e70334-s001.zip › acel70334-sup-0002-FigureS1-S2@Supplemental figure_2-01.pdf]

A

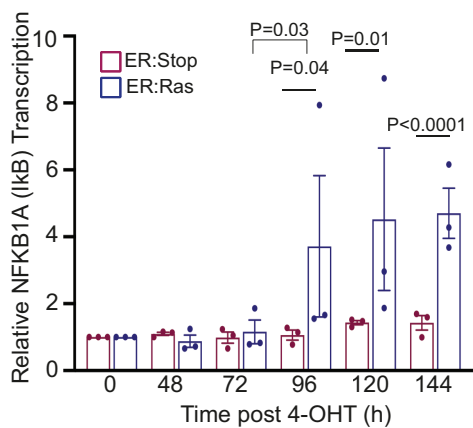

B

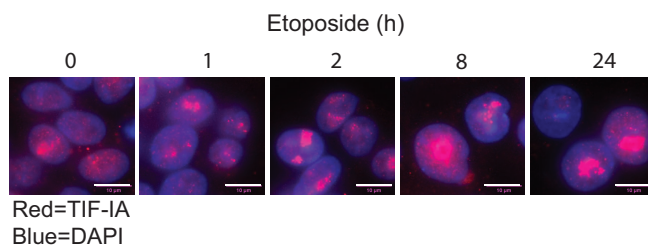

C

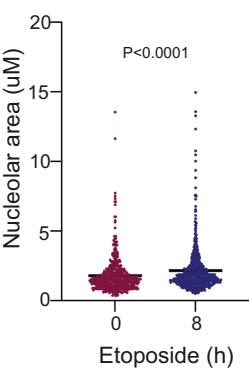

D

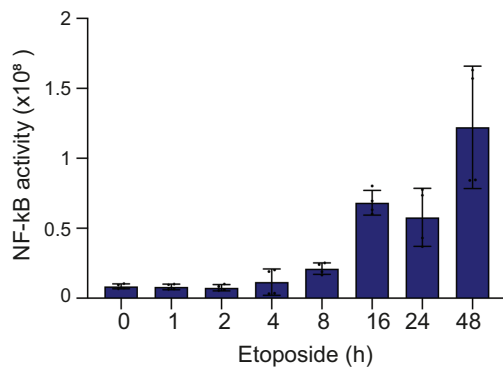

E

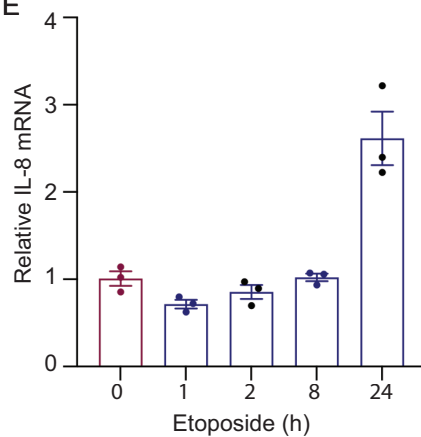

F

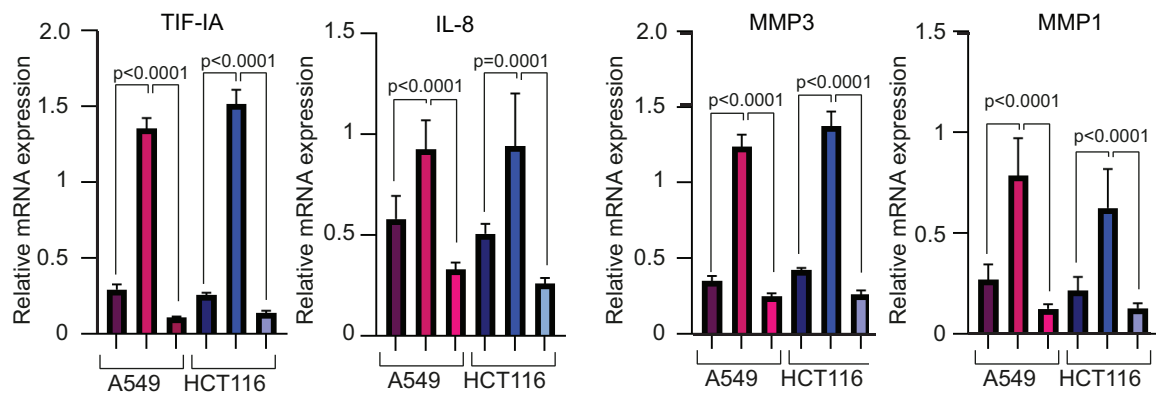

A549 SiCon DMSO (48h)  
 A549 SiCon Etop (48h)  
 A549 SiTIF#2 Etop (48h)  
 HCT SiCon DMSO (48h)  
 HCT SiCon Etop (48h)  
 HCT SiTIF#2 Etop (48h)

G

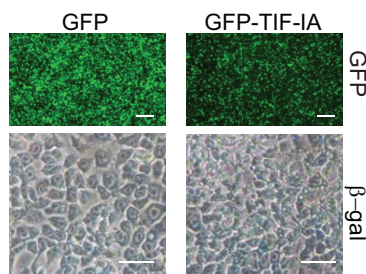

Supplement: Supplementary file 1 — Data S1: acel70334‐sup‐0001‐DataS1.zip. [file ACEL-25-e70334-s001.zip › acel70334-sup-0004-FigureS3@Supplemental figure_3RSB.pdf]

# Supplemental Figure 4

A

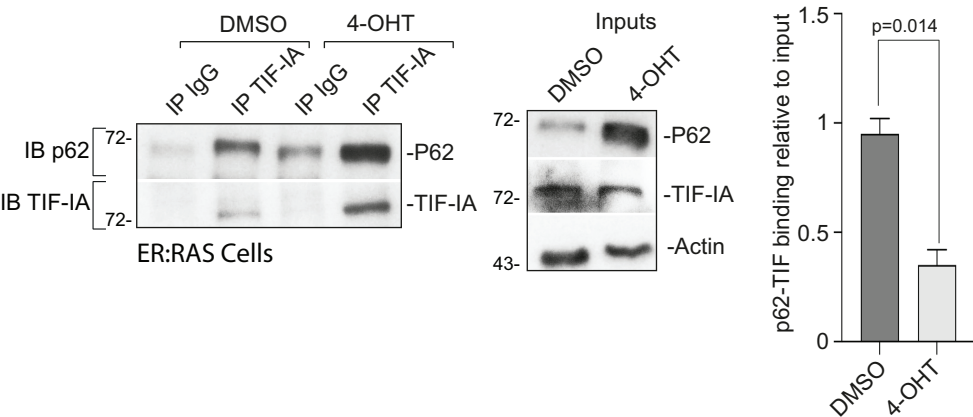

B

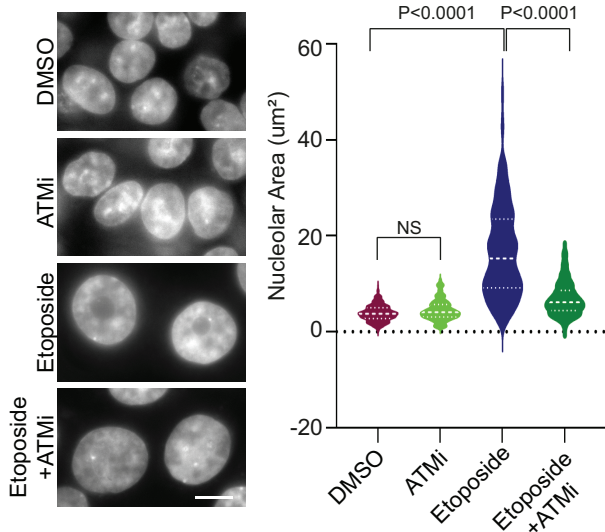

Supplement: Supplementary file 1 — Data S1: acel70334‐sup‐0001‐DataS1.zip. [file ACEL-25-e70334-s001.zip › acel70334-sup-0005-FigureS4.pdf]
